# Exploration of microRNA Biomarkers in Blood Small Extracellular Vesicles for Enzootic Bovine Leukosis

**DOI:** 10.3390/microorganisms11092173

**Published:** 2023-08-28

**Authors:** Akane Takada, Yuji O. Kamatari, Kaori Shimizu, Ayaka Okada, Yasuo Inoshima

**Affiliations:** 1 Laboratory of Food and Environmental Hygiene, Cooperative Department of Veterinary Medicine, Faculty of Applied Biological Sciences, Gifu University, 1-1 Yanagido, Gifu 501-1193, Japan; 2 Institute of Glyco-Core Research (iGCORE), Gifu University, 1-1 Yanagido, Gifu 501-1193, Japan; 3 The United Graduate School of Drug Discovery and Medical Information Sciences, Gifu University, 1-1 Yanagido, Gifu 501-1193, Japan; 4 Division of Instrumental Analysis, Life Science Research Center, Gifu University, 1-1 Yanagido, Gifu 501-1193, Japan; 5 Education and Research Center for Food Animal Health, Gifu University (GeFAH), 1-1 Yanagido, Gifu 501-1193, Japan; 6 Joint Graduate School of Veterinary Sciences, Gifu University, 1-1 Yanagido, Gifu 501-1193, Japan

**Keywords:** enzootic bovine leukosis, bovine leukemia virus, blood, biomarker, small extracellular vesicles, miRNA

## Abstract

Enzootic bovine leukosis (EBL) is a B-cell lymphosarcoma caused by the bovine leukemia virus (BLV). While most infected cattle show no clinical signs, approximately 30% of infected cattle develop persistent lymphocytosis (PL), and a small percentage may develop EBL. Currently, there is no method for predicting the possibility of EBL onset. In this study, we analyzed the microRNAs (miRNAs) encapsulated in small extracellular vesicles (sEVs) in the blood to explore the biomarkers of EBL. To identify candidate biomarkers, blood samples were collected from three BLV-uninfected and three EBL cattle. Total RNA was extracted from filtered serum and used for microarray analysis. Due to their association with cancer in human orthologs, we selected three miRNAs as candidate biomarkers, bta-miR-17-5p, bta-miR-24-3p, and bta-miR-210, which were more than twice as abundant in EBL cattle than in BLV-uninfected cattle. Quantitative real-time polymerase chain reaction (qPCR) using serum RNAs from six cattle used for the microarray analysis was carried out for the detection of the three selected miRNAs. Additionally, bta-miR-92a, whose ortholog has been associated with cancer in humans, was also examined by qPCR. bta-miR-17-5p, bta-miR-24-3p, and bta-miR-92a, were successfully detected, but bta-miR-210 was not. To further evaluate the utility of these three miRNAs as biomarkers, new blood samples were collected from 31 BLV-uninfected and 30 EBL cattle. The levels of bta-miR-17-5p, bta-miR-24-3p, and bta-miR-92a, were significantly higher in EBL cattle than in BLV-uninfected cattle. These results suggest that increased levels of bta-miR-17-5p, bta-miR-24-3p, and bta-miR-92a in the blood could be used as biomarkers for EBL. This study may contribute to the control of BLV infections and develop a prediction method of EBL onset.

## 1. Introduction

Bovine leukemia virus (BLV), which belongs to the genus *Deltaretrovirus* in the family *Retroviridae* [1], infects B cells and causes enzootic bovine leukosis (EBL). EBL is one of the listed infectious diseases in the Act on Domestic Animal Infectious Diseases Control in Japan. Cattle developing EBL present with clinical signs such as protruding eyeballs, slimming, enlarged superficial lymph nodes, and increased numbers of dysmorphic peripheral lymphocytes. Approximately 30% of BLV-infected cattle develop persistent lymphocytosis (PL), and most show no clinical signs [2]. Only 1% to 5% of BLV-infected cattle develop EBL and present with B-cell malignant lymphoma [3]. Commonly, the detection of BLV DNA and antibodies against BLV and evaluation of BLV proviral load are used for the diagnosis of BLV infection, as outlined in the Livestock Mutual Aid Office Handling Guidelines [4]. Although these methods can be used for diagnosis of BLV infection, they cannot predict the development of EBL.

In 2013, a nationwide survey conducted in Japan revealed that as high as 40.9% of dairy cattle and 28.7% of beef breeding cattle had antibodies against BLV [5]. Therefore, countermeasures using the approach of test-and-slaughter could not be applicable. Moreover, the number of EBL cases at slaughterhouses and farms is gradually increasing [6]. Since all cattle diagnosed with EBL are prohibited to use for milk or meat production by the Slaughterhouse Act, EBL onset causes significant economic losses for farmers and the livestock industry. At present, there is no method of treatment for EBL or vaccine against BLV. EBL is diagnosed at slaughterhouses by postmortem inspections and farms by clinical signs, as mentioned above. On farms, several EBL-diagnostic biomarkers in blood tests were reported previously, including high BLV copy numbers [7], high total lactate dehydrogenase (LDH) activity, increase ratios of LDH isozymes 2 and 3 [8], and high thymidine kinase (TK) [9], as well as clinical aspects. However, these criteria were not completely applicable to all EBL cattle, and there is no established reliable method for predicting EBL onset. To address this, we aimed to identify novel potential biomarkers for EBL.

Small extracellular vesicles (sEVs), membrane-bound vesicles of 40–150 nm in diameter, are present in almost all biological fluids [10,11], including blood [12], urine [13], pleural effusions [14], and breast milk [15]. sEVs encapsulate nucleic acids such as messenger RNAs (mRNAs), microRNAs (miRNAs), DNAs, and lipids and proteins [16]. Studies have reported that miRNAs in blood sEVs can promote tumorigenesis and metastasis in recipient cells of both humans and mice [17,18]. miRNAs are ~22-nucleotide, small noncoding RNA molecules that bind to the 3′-UTR of target mRNAs and regulate stability or translation efficiency [19,20]. In humans, miRNAs in blood sEVs have been reported to play functional roles by transferring to recipient cells and regulating the expression of target genes, and they have attracted attention as biomarkers for cancer diagnosis [21,22]. It was reported that miR-15a-5p levels are higher in the plasma sEVs of patients with endometrial cancer than in those of healthy controls, and miR-15a-5p may be a potential biomarker for the diagnosis of endometrial cancer [23]. However, in cattle, miRNAs have not yet been reported as disease biomarkers in blood sEVs. EBL is one of the blood cancers in cattle; therefore, miRNAs in sEVs could also serve as biomarkers for EBL.

As mentioned above, accurate biomarkers for EBL remain unidentified. In this study, to explore novel blood biomarkers for EBL, we compared miRNA profiles in blood sEVs obtained from three BLV-uninfected cattle and three EBL cattle using microarray analysis and selected miRNA biomarker candidates for EBL. We then evaluated the utility of the selected miRNA biomarker candidates using blood sEVs samples newly collected from 31 BLV-uninfected and 30 EBL cattle.

## 2. Materials and Methods

All animal experimental protocols were approved by the Gifu University Animal Care and Use Committee (approval numbers 17046 and 2019-234). 

### 2.1. Animals

A total of 67 Holstein dairy cattle from farms and slaughterhouses were used, and blood samples were collected (Supplementary Table S1). Cattle with EBL were diagnosed at the veterinary clinics of NOSAI Aichi (Aichi, Japan), NOSAI Chiba (Chiba, Japan), NOSAI Gifu (Gifu, Japan), NOSAI Hokkaido (Hokkaido, Japan), or Toyohashi City Meat Hygiene Inspection Center (Aichi, Japan) in Japan, as described below.

### 2.2. Diagnosis of EBL

At NOSAI Aichi, Chiba, Gifu, and Hokkaido, EBL was diagnosed on farms by various methods, as mentioned above, based on the Livestock Mutual Aid Office Handling Guidelines [4]. At the Toyohashi City Meat Hygiene Inspection Center, EBL was diagnosed by prebiopsy, pre-disassembly inspection, and post-disassembly inspection, based on the New Meat Hygiene Inspection Manual (National Meat Inspection Office Council, 2011).

### 2.3. Hematology

Total white blood cell (WBC) and lymphocyte counts were measured by a cell counter, Celltac α MEK-6550 (Nihon Kohden, Tokyo, Japan). Cattle with PL was determined by evaluating lymphocyte counts and age based on the European Community’s leukosis key, which is one of the detection methods for PL in cattle [24,25].

### 2.4. Detection of Serum Antibodies against BLV

Anti-BLV antibodies in serum were detected using an anti-BLV antibody enzyme-linked immunosorbent assay (ELISA) kit (JNC, Tokyo, Japan) according to the manufacturer’s instructions.

### 2.5. Detection of BLV Provirus by Nested PCR

Total DNA was extracted from the WBC using the DNeasy Blood & Tissue Kit (69506, Qiagen, Hilden, Germany). Nested PCR was performed to detect the pX [26] or envelope [27] regions of BLV in DNA extracted from the WBC, using GoTaq Hot Start Green Master Mix (M512C, Promega, Madison, WI, USA). The thermal cycling conditions were as follows: an initial denaturation step at 94 °C for 9 min, followed by 25 cycles for denaturation at 94 °C for 45 s, annealing at 62 °C for 30 s, amplification at 72 °C for 30 s, and final elongation at 72 °C for 4 min.

### 2.6. Measurement of BLV Proviral Load by Quantitative Real-Time Polymerase Chain Reaction (qPCR)

The BLV proviral load (copies/10^5^ WBC) was estimated using the coordination of common motifs (CoCoMo)-BLV Primer/Probe (A803; RIKEN Genesis, Tokyo, Japan) according to the manufacturer’s instructions.

The hematological tests, detection of serum antibodies against BLV, and measurement of BLV proviral load were performed by the Gifu Chuo Livestock Hygiene Service Center (Gifu, Japan).

### 2.7. LDH Analysis

Total LDH activity (IU/l) was measured by an autoanalyzer JCA-BM6050 (JEOL, Tokyo, Japan) using enzymatic method L-Type Wako LD IF or L-Type Wako J (FUJIFILM Wako Pure Chemical Corporation, Osaka, Japan). The percentages of LDH isozymes were measured using a Hydrasys 2 Scan (Sebia, Paris, France) with a HYDRAGEL 7 ISO-LDH (Sebia). 

Measurements of total LDH activity and LDH isozymes were performed by a clinical laboratory testing company, Fujifilm Vet Systems (Tokyo, Japan).

### 2.8. Isolation and Characterization of Blood sEVs

We isolated the sEVs by sequentially filtrating the sera through 1.0, 0.45, and 0.2 μm pore-size filters (SLAP02550, SLHVR33RS, and SLGVR33RS, Merck Millipore, Cork, Ireland). 

Isolated sEVs were biophysically characterized by transmission electron microscopy (TEM), nanoparticle size distribution analysis, and Western blot (WB), based on the Minimal Information for Studies of Extracellular Vesicles 2018 (MISEV2018) guidelines [28]. For TEM, sEVs were purified using filtered sera and a miRCURY Exosome Serum/Plasma Kit (76603, Qiagen), according to the manufacturer’s instructions. Purified sEVs were diluted 1:100 with 0.1 μm filtered PBS and then filtered again sequentially, as described above. Following filtration, the sEVs suspension was applied to glow-discharged carbon support films on a copper grid (U1013, EM Japan, Tokyo, Japan). The grids were stained with uranyl acetate and were examined under a JEM-2100F electron microscope (JEOL, Tokyo, Japan) at 200 kV. For the nanoparticle size analysis, the sEVs suspension was prepared in a similar manner to the TEM sample preparation. Nanoparticle size distribution analysis was performed using a VIDEO DROP nanoparticle imaging analyzer (Myriade, Paris, France). For WB, isolated sEVs were confirmed by anti-CD63 and anti-Flotillin-1 antibodies, as described previously [29,30]. The sEVs suspension was diluted 1:100, and 50 μL of diluted samples was then mixed with 50 μL of 2 × sodium dodecyl sulfate (SDS) sample buffer (0.1 M Tris-HCl, pH6.8, 12% 2-mercaptoethanol (*v*/*v*), 4% SDS (*w*/*v*), 20% glycerol (*v*/*v*), and 1% bromophenol blue (*w*/*v*)) and boiled for 5 min at 95 °C before electrophoresis. We used anti-CD63 (1:1000, sc-31214; Santa Cruz Biotechnology, Dallas, TX, USA) and anti-Flotillin-1 (1:100, 610820; BD Transduction Laboratories, Franklin Lakes, NJ, USA) antibodies as primary antibodies. Donkey antigoat IgG, F(ab’)_2_–HRP (1:2000, sc-3851, Santa Cruz Biotechnology) and antimouse IgG, HRP-linked (1:2000, 7076BC, Cell Signaling Technology, Danvers, MA, USA) antibodies were used as secondary antibodies.

### 2.9. Microarray Analysis

To explore biomarker candidates for EBL, blood samples were collected from three BLV-uninfected cattle (Nos. 1–3) and three cattle with EBL (Nos. 4–6) (Supplementary Table S1). Total RNA extraction, microarray analysis, and data normalization were performed by an analysis service company, Filgen (Nagoya, Japan). Total RNA was extracted from filtered serum using the miRNeasy Micro Kit (217084, Qiagen) according to the manufacturer’s instructions with slight modifications. The concentration and quality of the extracted total RNA were estimated using the NanoDrop One (ND-ONE-W, Thermo Fisher Scientific, Waltham, MA, USA). For microarray analysis, we used the GeneChip miRNA 4.0 Array (902412, Affymetrix, Santa Clara, CA, USA), which contains probes for 30,424 mature miRNAs from 203 organisms, including viruses on the slide. In the array for 30,424 target miRNAs, probes for 783 bovine miRNAs are included. The hybridized microarray slides were scanned, and fluorescence intensities were measured using a GeneChip Scanner 3000 7G (GCS3000-01, Thermo Fisher Scientific). The obtained data were analyzed and normalized by robust multiarray average and detection above background algorithms (RMA-DABG) using the Expression Console Software version 1.4.1.46 (Affymetrix, Thermo Fisher Scientific). After normalization according to the manufacturer’s instructions, the differential levels of miRNAs between BLV-uninfected and EBL cattle were examined. The corrected *p*-value cutoff was set at 0.05, and miRNAs with significance between BLV-uninfected cattle and EBL cattle were selected as biomarker candidates for EBL.

### 2.10. Quantification of Levels of miRNA Biomarker Candidates for EBL in sEVs by qPCR

To quantify the levels of miRNA biomarker candidates for EBL in sEVs, we used three BLV-uninfected (Nos. 1–3) and three EBL cattle (Nos. 4–6) (Supplementary Table S1). Total RNA was extracted from the sEVs as described in the microarray analysis section. The concentration and quality of the extracted total RNA were estimated using a NanoDrop Lite (ND-LITE-PR, Thermo Fisher Scientific). We synthesized cDNA from 6 μL of 5 ng/μL of extracted total RNA using miRCURY LNA RT Kit (339340, Qiagen) according to the manufacturer’s instructions and used it for qPCR. Quantification of the miRNA biomarker candidates was performed using the miRCURY LNA SYBR Green PCR Kit (339346, Qiagen). Primers for gga-miR-17-5p (YP00205960, Qiagen), hsa-miR-24-3p (YP00204260, Qiagen), cfa-miR-210 (YP02119434, Qiagen), and hsa-miR-92a-3p (YP00204258, Qiagen) were contained in miRCURY LNA PCR Assay components (339306, Qiagen). The detailed sequence information of primers is provided in Supplementary Table S3. For qPCR, the cDNA was diluted at 1:30 by adding RNase-free water, and 3 μL of diluted cDNA was used in a total reaction volume of 10 μL. We performed qPCR as described previously [30]. Because suitable internal control miRNAs encapsulated in bovine blood sEVs have not been identified yet, we evaluated miRNA amounts in blood sEVs using Ct values.

### 2.11. Validation of miRNA Biomarker Candidates for EBL in Blood sEVs

After the selection of the miRNA biomarker candidates for EBL by microarray analysis, new blood samples were collected from 31 BLV-uninfected cattle (Nos. 7–37) and 30 EBL cattle (Nos. 38–67) to evaluate the utility of miRNA biomarker candidates for EBL (Supplementary Table S1). We isolated the sEVs from the sera of cattle Nos. 7–11, as well as 38 (Supplementary Table S1) by sequential filtration through 1.0, 0.45, and 0.2 μm pore-size filters (SLAP02550, SLHVR33RS, and SLGVR33RS, Merck Millipore). Sera from cattle Nos. 12–37 and 39–67 (Supplementary Table S1) were filtered sequentially through 1.0 μm (SFGF013100N, Membrane Solutions, Auburn, WA, USA), 0.45 μm (6791-1302, Cytiva, Tokyo, Japan) and 0.2 μm (S-1304, KURABO, Osaka, Japan) pore-size filters. After filtration, the sEVs were purified using the miRCURY Exosome Serum/Plasma Kit according to the manufacturer’s instructions. Purified sEVs were used to validate miRNA biomarker candidates for EBL using qPCR, similar to the method used for the quantification of miRNA biomarker candidates. 

### 2.12. Statistical Analysis

The data were analyzed for significance using Mann–Whitney U test. Correlation coefficients were analyzed using Spearman’s rank correlation or Pearson’s correlation. The corrected *p*-value cutoff was set at 0.05.

## 3. Results

### 3.1. BLV Infection and Clinical Status

**Figure 1:**
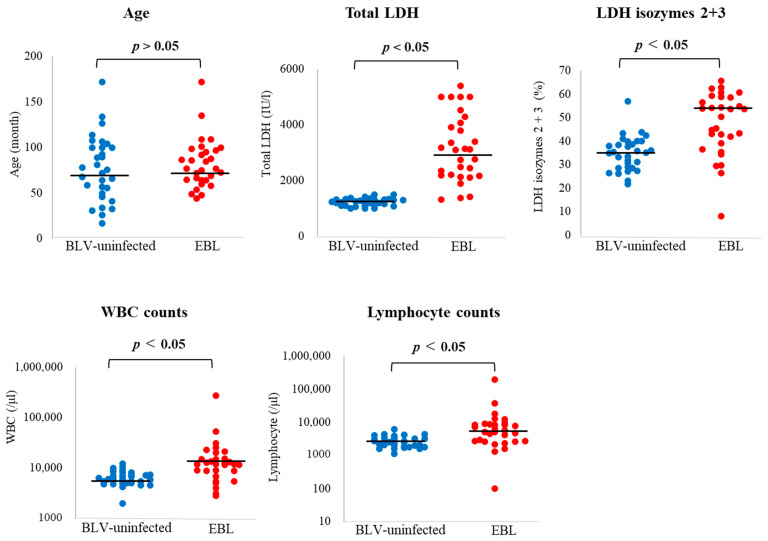
The significant differences between bovine leukemia virus (BLV)-uninfected cattle (*n* = 31) and enzootic bovine leukosis (EBL) cattle (*n* = 30) for several diagnostic criteria of EBL: Regarding age, there was no significance between BLV-uninfected cattle and EBL cattle. Regarding total lactate dehydrogenase (LDH), LDH isozymes 2 + 3, white blood cell (WBC) counts, and lymphocyte counts, there was significance between BLV-uninfected cattle and EBL cattle. The significance between the two groups was determined using Mann–Whitney U test. The median of each group is displayed as a horizontal bar. IU, international unit.

Data on BLV infection, the clinical status of cattle used in the microarray analysis, and the validation of miRNA biomarker candidates for EBL are summarized in Supplementary Table S1. Significant differences between BLV-uninfected and EBL cattle in several diagnostic criteria of EBL were examined, including age, total LDH, LDH isozymes 2 + 3, WBC and lymphocyte counts. There was no significant difference in age between BLV-uninfected and EBL cattle, whereas there was a significant difference in total LDH, LDH isozyme 2 + 3, lymphocyte counts, and WBC counts between BLV-uninfected and EBL cattle (Figure 1).

### 3.2. Confirmation of sEVs 

**Figure 2:**
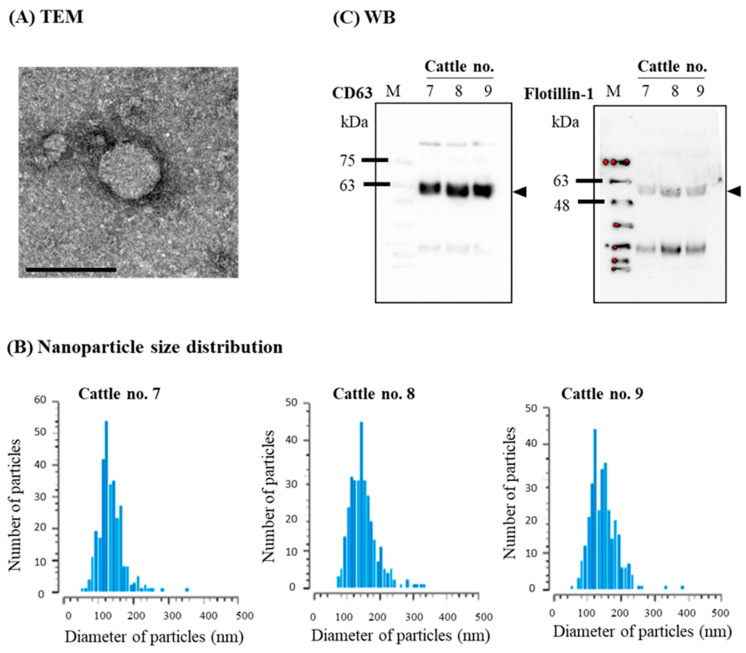
Confirmation of isolated blood small extracellular vesicles (sEVs): (**A**) Transmission electron microscopy (TEM) analysis showed the bilayer spherical shape of sEVs (scale bar shows 100 nm). (**B**) Nanoparticle size distribution of blood sEVs. Nanoparticle size distribution of isolated sEVs from each bovine leukemia virus (BLV)-uninfected cattle was measured repetitively. (**C**) Western blot (WB) analysis using antibodies against sEV surface and internal marker proteins, CD63 and flotillin-1, respectively.

The morphology of the isolated sEVs was observed byTEM, which revealed a round shape (Figure 2A). Nanoparticle size distribution analysis showed that the peak distribution was approximately 100–150 nm for all samples (Figure 2B). Furthermore, the sEV surface marker protein CD63 and an internal protein, flotillin-1, were detected using WB analysis (Figure 2C, arrow heads), confirming the successful isolation of sEVs. 

### 3.3. Microarray Analysis

To explore miRNA biomarker candidates for EBL, microarray analysis was conducted to determine the species and amounts of miRNAs in blood sEVs derived from three BLV-uninfected cattle (Nos. 1–3) and three EBL cattle (Nos. 4–6) (Supplementary Table S2). Among 783 bovine miRNAs in the array, 235 miRNAs and 255 miRNAs were detected in all three cattle in BLV-uninfected and in EBL groups, respectively. We selected the miRNAs that were more than two-fold higher in levels in EBL cattle than in BLV-uninfected cattle, resulting in a reduction of 63 miRNAs for subsequent analysis. Differences in miRNAs levels between the three BLV-uninfected cattle and three EBL cattle were determined using Mann–Whitney U test. The miRNAs with a corrected *p*-value of less than 0.05 were considered significantly fluctuating miRNAs encapsulated in blood sEVs, resulting in a reduction in the number to seven (bta-miR-17-5p, bta-miR-24-3p, bta-miR-210, bta-miR-2305, bta-miR-2343, bta-miR-2413, and bta-miR-2422). Among these seven miRNAs, bta-miR-17-5p, bta-miR-24-3p, and bta-miR-210, whose orthologs are associated with cancer in humans [22,31,32], were selected as candidate biomarkers for EBL. Ultimately, the three miRNAs (bta-miR-17-5p, bta-miR-24-3p, and bta-miR-210) were selected as potential biomarker candidates for EBL and used for further analysis. 

### 3.4. qPCR for Detection of miRNA Biomarker Candidates for EBL in sEVs 

**Figure 3:**
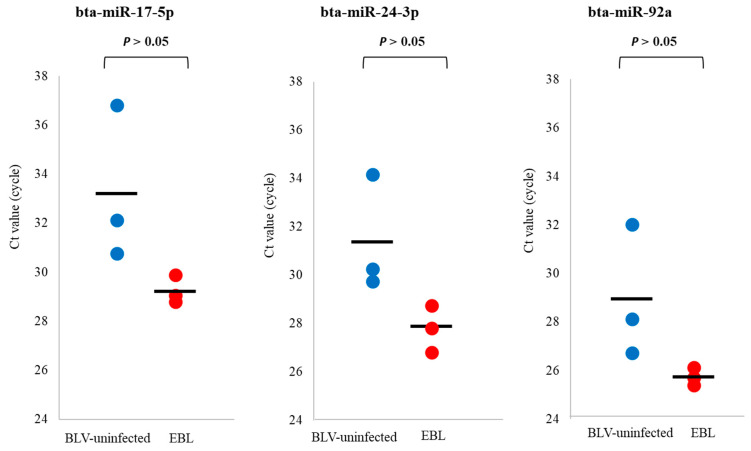
Cycle threshold (Ct) values of bovine leukemia virus (BLV)-uninfected cattle (*n* = 3) and enzootic bovine leukosis (EBL) cattle (*n* = 3) used in microarray analysis: The levels of bta-miR-17-5p, bta-miR-24-3p, and bta-miR-92a used in microarray analysis tended to be higher but not significant in blood small extracellular vesicles (sEVs) from EBL cattle than in those from BLV-uninfected cattle. The significance between the two groups was determined using Mann–Whitney U test. The mean of Ct values is displayed as a horizontal bar.

qPCR was carried out to confirm whether the three selected miRNAs could be used as biomarkers for EBL. The three biomarker candidates for EBL were validated by qPCR using the extracted total RNAs used in the microarray analysis. In addition, bta-miR-92a was also used for qPCR, as it is reported to be associated with cancer in human ortholog [22,33], although it did not satisfy the above selection criteria. The levels of bta-miR-17-5p and bta-miR-24-3p tended to be higher in sEVs from EBL cattle than in those from BLV-uninfected cattle, in accordance with the microarray results. We also detected bta-miR-92a by qPCR, and its levels were higher in sEVs from EBL cattle than in those from BLV-uninfected cattle (Figure 3). However, bta-miR-210 was not detected by qPCR, possibly because its level in sEVs was below the detection limit of qPCR, or the sequence of primers used for the detection of bta-miR-210 did not partially match the sequence of the gene. Thus, only bta-miR-17-5p, bta-miR-24-3p, and bta-miR-92a were selected as potential biomarkers for EBL.

### 3.5. qPCR for Evaluation of the Utility of miRNA Biomarker Candidates for EBL

**Figure 4:**
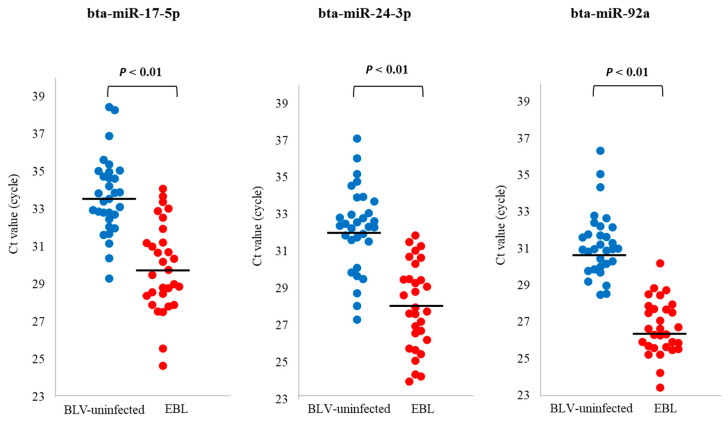
Cycle threshold (Ct) values of bovine leukemia virus (BLV)-uninfected cattle (*n* = 31) and enzootic bovine leukosis (EBL) cattle (*n* = 30): The levels of bta-miR-17-5p, bta-miR-24-3p, and bta-miR-92a were significantly higher in blood small extracellular vesicles (sEVs) from newly collected EBL cattle than in those from newly collected BLV-uninfected cattle. The significance between the two groups was determined using Mann–Whitney U test. The median of the Ct values is displayed as a horizontal bar.

To validate the three miRNAs, bta-miR-17-5p, bta-miR-24-3p, and bta-miR-92a, as biomarker candidates for EBL, qPCR was carried out using newly collected blood sEVs from 31 BLV-uninfected and 30 EBL cattle (Supplementary Table S1). The levels of bta-miR-17-5p, bta-miR-24-3p, and bta-miR-92a were significantly higher in sEVs from EBL cattle than in those from BLV-uninfected cattle (Figure 4).

### 3.6. Correlations between Levels of miRNA Biomarker Candidates for EBL in sEVs and Several Diagnostic Criteria of EBL

Correlations between the levels of miRNA biomarker candidates for EBL in sEVs and several diagnostic criteria for EBL were examined, including the age of cattle, BLV proviral load, total LDH, LDH isozymes 2 + 3, WBC count, and lymphocyte count (Supplementary Figures S1–S6). Age and BLV proviral load were not related to the levels of miRNA biomarker candidates for EBL in sEVs. Total LDH, LDH isozymes 2 + 3, WBC counts, and lymphocyte counts were related to the levels of miRNA biomarker candidates for EBL in sEVs.

## 4. Discussion

In this study, we aimed to explore miRNA biomarkers of EBL in blood sEVs. We revealed that the levels of three miRNAs, bta-miR-17-5p, bta-miR-92a, and bta-miR-24-3p, were higher in sEVs from EBL cattle than in those from BLV-uninfected cattle, suggesting that these miRNAs could be used as biomarkers for EBL.

In humans, hsa-miR-17-5p and hsa-miR-92a-3p belong to the miR-17/92 cluster [34], which is overexpressed in B-cell lymphoma [35,36]. The miR-17/92 cluster suppresses the apoptosis-inducing protein Bim and tumor suppressor proteins p21 and PTEN [37,38,39]. Furthermore, hsa-miR-17-5p suppresses the transcription factor E2F1 via cell cycle repression [40]. Since bta-miR-17-5p differs by only one mer from the sequence of hsa-miR-17-5p, and bta-miR-92a has the same sequence as hsa-miR-92a-3p (Supplementary Table S4), these miRNAs may also suppress tumor suppressor proteins, such as Bim, p21, and E2F1, and thereby promote the progression of EBL.

In Hodgkin lymphoma in humans, it has been reported that hsa-miR-24-3p suppress the tumor suppressor protein p27, the transcription factor c-Myc, which has cell cycle repressive effects, and apoptosis-inducing protein DEDD [41,42]. Since bta-miR-24-3p has the same sequence as hsa-miR-24-3p (Supplementary Table S4), bta-miR-24-3p may also suppress tumor suppressor proteins such as p27, c-Myc, and DEDD, thereby promoting the progression of EBL. In humans, it has been reported that sEVs carrying miRNA biomarkers for cancer are secreted from tumor cells [43]. Although we have not clarified whether the miRNA biomarker candidates are secreted from tumor cells or from cells that have not yet become tumors, sEVs carrying the miRNA biomarker candidates selected in this study may also be secreted from tumor cells (B cell lymphoma). Therefore, the miRNA candidate biomarkers may promote tumor progression by negatively regulating the expression of tumor suppressor proteins. However, further validation of these findings is required.

Cattle that develop EBL are generally older than 3–5 years [2,44]. However, in recent years, in Japan, some EBL cases have been reported in young Japanese Black beef cattle as well [45,46]. Therefore, to demonstrate whether miRNA biomarker candidates for EBL can be used regardless of age, we analyzed the correlation between the level of miRNAs and cattle age. There was no relationship between the levels of the three candidate miRNAs and cattle age, suggesting that the level of miRNAs was not influenced by age. Therefore, the miRNA biomarker candidates selected in this study can be useful for both adult and young EBL cattle. Furthermore, the relationship between the levels of miRNAs and EBL diagnostic criteria reported previously was also evaluated. However, there was no relationship between the level of miRNA biomarker candidates and the BLV proviral load. This indicates that viral infection and viral copy numbers are not the only reasons for the development or onset of EBL, consistent with previous reports that EBL can occur in cattle even when the BLV proviral load is low [7,47]. There was a relationship between the level of miRNA biomarker candidates selected in this study and total LDH, LDH isozymes 2 + 3, and WBC and lymphocyte counts. Total LDH and LDH isozymes 2 + 3 have been reported to be diagnostic markers for EBL [8]. Moreover, increased WBC and lymphocyte counts tend to be correlated with EBL [48]. The correlation between the levels of miRNAs and EBL diagnostic criteria, such as total LDH, LDH isozymes 2 + 3, and WBC and lymphocyte counts, indicates that the miRNA biomarker candidates in this study could be used as novel biomarkers for EBL. In combination with previous diagnostic criteria, these novel biomarkers can provide a more reliable diagnosis of EBL. All of these tests, including quantification of miRNA biomarkers, LDH, and WBC and lymphocyte counts, can be performed using a single blood sample, making this technology easy to introduce and helpful. Because this study used EBL cattle samples, and the sample number for evaluation of selected biomarkers is limited, further investigations using pre-EBL samples, as well as blood samples from cattle with other disease conditions or other infections, are needed to evaluate their utility as predictive biomarkers. Moreover, the levels of selected biomarkers from some EBL cattle were overlapped with a part of those from BLV-uninfected cattle, suggesting that diagnostic sensitivity and specificity of the selected biomarkers should be improved by the combination with other criteria of EBL diagnosis.

In previous studies, hsa-miR-424-5p was significantly increased in milk sEVs from BLV-infected cattle with high proviral loads and EBL cattle [29,30]. However, in this study, the results of microarray analysis show that hsa-miR-424-5p was not a biomarker candidate for EBL in blood sEVs, suggesting that miRNAs encapsulated in milk sEVs are different from those in blood sEVs. Future validation with blood and milk samples from the same cattle is required to confirm the differences in miRNAs encapsulated in sEVs.

In conclusion, we found that the levels of bta-miR-17-5p, bta-miR-24-3p, and bta-miR-92a in blood sEVs from cattle with EBL were higher than those from BLV-uninfected cattle, and these three miRNAs could be potential biomarkers for EBL. This study may contribute to further research exploring predictive biomarkers for EBL in cattle and to controlling EBL.

## Data Availability

The data presented in this study are available within the article and in the Supplementary Materials.

## Supplementary Materials

The following supporting information can be downloaded at: https://www.mdpi.com/article/10.3390/microorganisms11092173/s1, Figure S1: Correlation between age and cycle threshold (Ct) values for biomarker candidates; Figure S2: Correlation between bovine leukemia virus (BLV) proviral load and cycle threshold (Ct) values for biomarker candidates; Figure S3: Correlation between total lactate dehydrogenase (LDH) and cycle threshold (Ct) values for biomarker candidates; Figure S4: Correlation between lactate dehydrogenase (LDH) isozymes 2 + 3 and cycle threshold (Ct) values for biomarker candidates; Figure S5: Correlation between white blood cell (WBC) counts and cycle threshold (Ct) values for biomarker candidates; Figure S6: Correlation between lymphocyte counts and cycle threshold (Ct) values for biomarker candidates; Table S1: BLV infection and clinical status of cattle used in this study; Table S2: Raw data of microarray analysis; Table S3: Primers used for qPCR; Table S4: Sequences of microRNAs.
