# Supplementary material for: Exploration of microRNA Biomarkers in Blood Small Extracellular Vesicles for Enzootic Bovine Leukosis
Source: Microorganisms. 2023 Aug 28;11(9):2173. doi: 10.3390/microorganisms11092173 (PMC10535767; doi:10.3390/microorganisms11092173)
Supplement: Supplementary file 1 [file microorganisms-11-02173-s001.zip › Supplementary Table S3,S4, Fig S1-S6.pdf]

Supplement to: **Exploration of microRNA biomarkers in blood small extracellular vesicles for enzootic bovine leukosis**

Akane Takada[1], Yuji O. Kamatari[2, 3, 4], Kaori Shimizu[1], Ayaka Okada[1, 5], Yasuo Inoshima[1, 5, 6, *]

Supplementary information includes four supplementary tables (Supplementary Tables S1 and S2 are shown as separate Excel files), and six supplemental figures in this file.

Supplementary Table S1. BLV infection and clinical status of cattle used in this study. Please see Supplementary Table S1 as an Excel file.
Supplementary Table S2. Raw data of microarray analysis. Please see Supplementary Table S2 as an Excel file.

Supplementary Table S3. Primers used for qPCR[a]

Primers[b]	Target genes	Sequence (5'-3')[c]	Accession number[c]	
			Primer genes	Target genes
gga-miR-17-5p	bta-miR-17-5p	CAAAGUGCUUACAGUGCAGGUAGU	MIMAT0001114	MIMAT0003815
hsa-miR-24-3p	bta-miR-24-3p	UGGCUCAGUUCAGCAGGAACAG	MIMAT0000080	MIMAT0003840
cfa-miR-210	bta-miR-210	ACUGUGCGUGUGACAGCGGCUGA	MIMAT0009846	MIMAT0003824
hsa-miR-92a-3p	bta-miR-92a	UAUUGCACUUGUCCCGGCCUGU	MIMAT0000092	MIMAT0009383

[a] qPCR, quantitative real-time polymerase chain reaction

[b] Primers were purchased from QIAGEN (product nos., YP00205960, YP00204260, YP02119434, and YP00204258).

[c] Obtained from miRbase (https://www.mirbase.org/search.shtml, accessed on 6 October, 2022)

Supplementary Table S4. Sequences of microRNAs

Target genes	Sequence (5'-3')	Accession number[a]
bta-miR-17-5p	CAAAGUGCUUACAGUGCAGGUAGU	MIMAT0003815
hsa-miR-17-5p	CAAAGUGCUUACAGUGCAGGUAG	MIMAT0000070
bta-miR-92a	UAUUGCACUUGUCCCGGCCUGU	MIMAT0009383
hsa-miR-92a-3p	UAUUGCACUUGUCCCGGCCUGU	MIMAT0000092
bta-miR-24-3p	UGGCUCAGUUCAGCAGGAACAG	MIMAT0003840
hsa-miR-24-3p	UGGCUCAGUUCAGCAGGAACAG	MIMAT0000080
bta-miR-210	ACUGUGCGUGUGACAGCGGCUGA	MIMAT0003824
has-miR-210-3p	CUGUGCGUGUGACAGCGGCUGA	MIMAT0000267

[a] Obtained from miRbase (https://www.mirbase.org/search.shtml, accessed on 28 October, 2022)

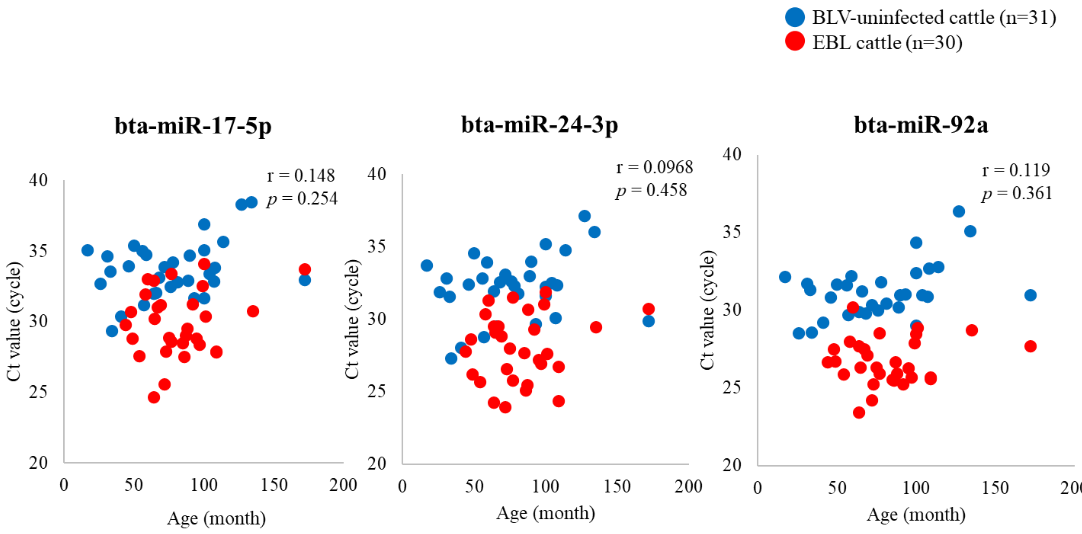

Supplementary Figure S1. Correlation between age and cycle threshold (Ct) values for biomarker candidates. There was no relation between age and Ct values for biomarker candidates in sEVs. The correlation between two continuous variables was detected using Pearson correlation analysis.

BLV, bovine leukemia virus. EBL, enzootic bovine leukosis.

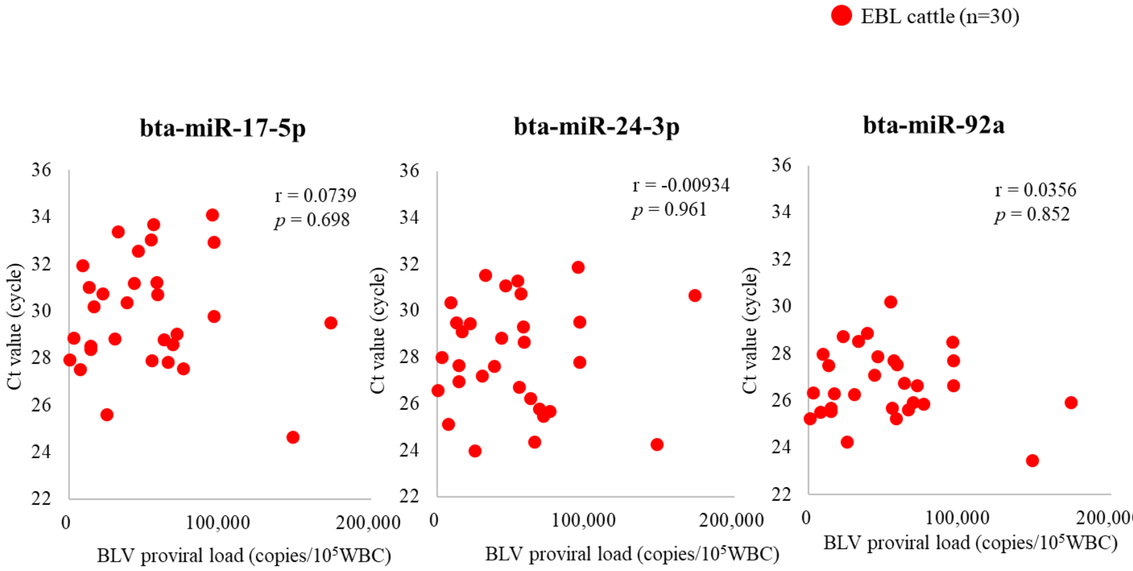

Supplementary Figure S2. Correlation between bovine leukemia virus (BLV) proviral load and cycle threshold (Ct) values for biomarker candidates. There was no relation between BLV proviral load and Ct values for biomarker candidates in sEVs. The correlation between two continuous variables was detected using Spearman's rank correlation analysis.

EBL, enzootic bovine leukosis. WBC, white blood cells.

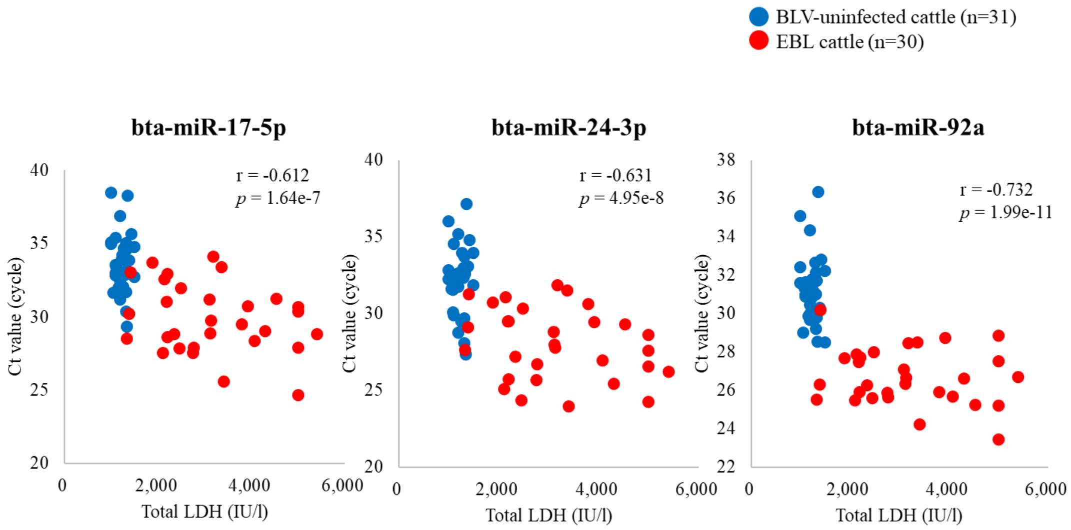

Supplementary Figure S3. **Correlation between total lactate dehydrogenase (LDH) and cycle threshold (Ct) values for biomarker candidates.** There was a significant difference between total LDH and Ct values for biomarker candidates in sEVs. The correlation between two continuous variables was detected using Spearman’s rank correlation analysis.

BLV, bovine leukemia virus. EBL, enzootic bovine leukosis. IU, international unit.

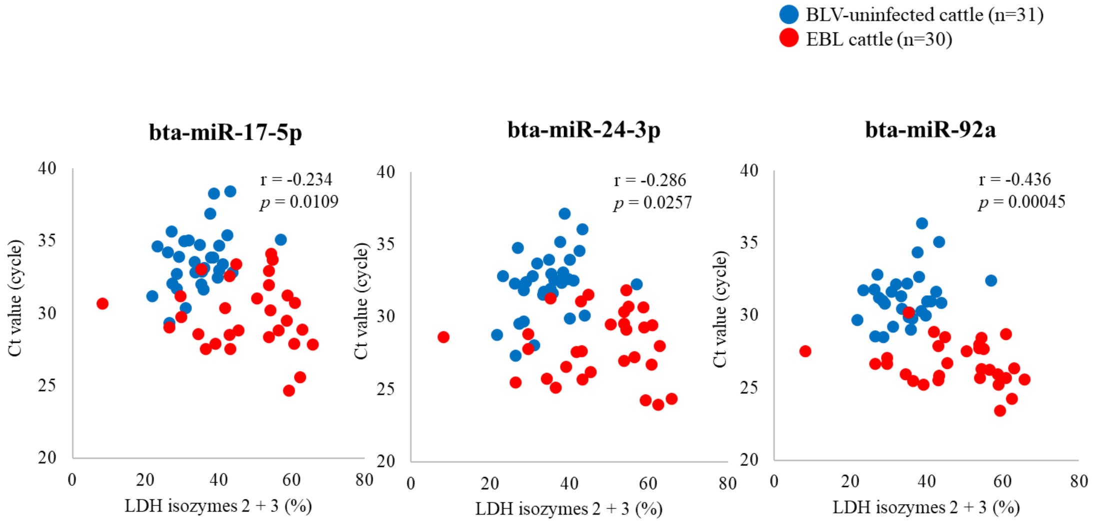

Supplementary Figure S4. **Correlation between lactate dehydrogenase (LDH) isozymes 2 + 3 and cycle threshold (Ct) values for biomarker candidates.** There was a significant difference between LDH isozymes2 + 3 and Ct values for biomarker candidates in sEVs. The correlation between two continuous variables was detected using Pearson correlation analysis.

BLV, bovine leukemia virus. EBL, enzootic bovine leukosis.

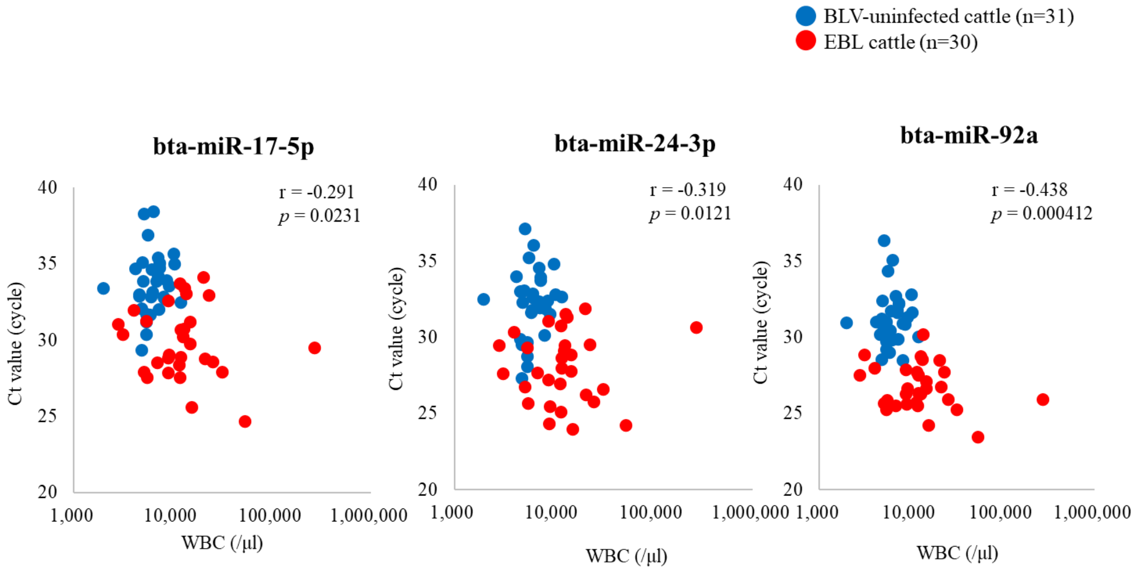

Supplementary Figure S5. Correlation between white blood cell (WBC) counts and cycle threshold (Ct) values for biomarker candidates. There was a significant difference between WBC counts and Ct values for biomarker candidates in sEVs. The correlation between two continuous variables was detected using Spearman's rank correlation analysis.
BLV, bovine leukemia virus. EBL, enzootic bovine leucosis.

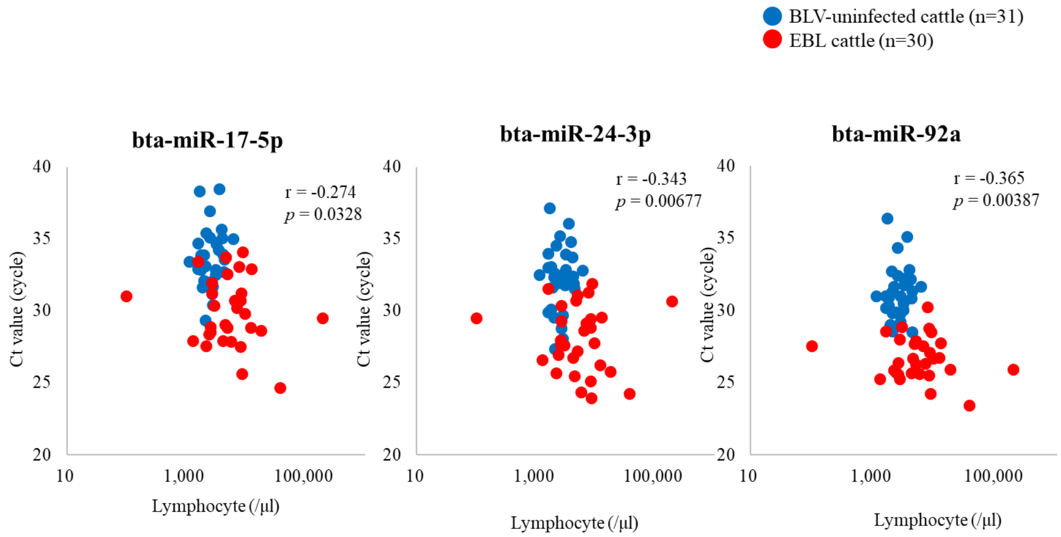

Supplementary Figure S6. Correlation between lymphocyte counts and cycle threshold (Ct) values for biomarker candidates. There was a significant difference between lymphocyte counts and Ct values for biomarker candidates in sEVs. The correlation between two continuous variables was detected using Spearman's rank correlation analysis.
BLV, bovine leukemia virus. EBL, enzootic bovine leukosis.
